# Spectral Deconvolution for Gas Chromatography Mass Spectrometry-Based Metabolomics: Current Status and Future Perspectives

**DOI:** 10.5936/csbj.201301013

**Published:** 2013-06-28

**Authors:** Xiuxia Du, Steven H Zeisel

**Affiliations:** a Department of Bioinformatics, University of North Carolina at Charlotte, Charlotte, NC, United States; b Nutrition Research Institute, University of North Carolina at Chapel Hill, Kannapolis, NC, United States

**Keywords:** Metabolomics, mass spectrometry, bioinformatics

## Abstract

Mass spectrometry coupled to gas chromatography (GC-MS) has been widely applied in the field of metabolomics. Success of this application has benefited greatly from computational workflows that process the complex raw mass spectrometry data and extract the qualitative and quantitative information of metabolites. Among the computational algorithms within a workflow, deconvolution is critical since it reconstructs a pure mass spectrum for each component that the mass spectrometer observes. Based on the pure spectrum, the corresponding component can be eventually identified and quantified. Deconvolution is challenging due to the existence of co-elution. In this review, we focus on progress that has been made in the development of deconvolution algorithms and provide thoughts on future developments that will expand the application of GC-MS in metabolomics.

## Introduction

Metabolomics is the comprehensive qualitative and/or quantitative study of a metabolome (the set of metabolites synthesized by an organism, tissue, or cells) and as such provides measurements essential for systems biology approaches for the study of health and disease. It has benefited greatly from advances in analytical technologies including nuclear magnetic resonance (NMR) and mass spectrometry (MS) coupled to separation techniques. The advantages of NMR are the minimal requirements for sample preparation and the non-discriminating and non-destructive nature of the technique. However, the low sensitivity of NMR makes it difficult to detect low-abundance metabolites that could constitute key discoveries of new biomarkers or biological mechanisms. Mass spectrometry-based metabolomics offers high selectivity and sensitivity and, more importantly, the potential to identify metabolites. Combining MS with separation techniques reduces the complexity of the mass spectra due to metabolite separation in the time dimension and provides additional information about the physical and chemical properties of the metabolites [1, 2]. Due to these advantages, the MS-based metabolomics approach is being widely used in food and nutrition research [3], plant science [4], marine science [5], environmental science [6], drug development and toxicology studies [7, 8], and many others.

Obtaining a full coverage of the metabolome generally requires multiple separation approaches since metabolites are heterogeneous, low molecular-weight components (less than 1,500 Da) that are characterized by a wide variation in physical and chemical properties (e.g., polarity, volatility, and solubility) [9]. Three types of separation techniques are commonly used in MS-based metabolomics: liquid chromatography (LC), gas chromatography (GC), and capillary electrophoresis (CE).

GC combined with electron ionization (EI) mass spectrometry allows for the identification and quantification of volatile and thermally stable compounds of low polarity from sources such as biological tissues and foods [10]. The fragmentation of metabolites during EI are highly characteristic of the chemical structure, allowing these mass spectra to be used for identification of compounds from mass spectral libraries [11]. Due to its high reproducibility, chromatographic peak resolution, and the existence of libraries of mass spectra, GC-EI-MS is regarded as the gold standard for metabolomics research [12]. However, GC-MS is incompatible with nonvolatile and thermally labile compounds. As a result, derivatization methods have been developed to make these metabolites less polar, more volatile and/or thermally stable so that they can be analyzed on GC-MS.

Complementary to GC-MS, LC-MS enables identification and quantification of high polarity compounds including organic acids, fatty acids, amino acids, and steroids. LC-MS-based metabolomics is generally performed using soft ionization techniques such as electrospray ionization (ESI) and atmospheric pressure chemical ionization (APCI), which do not cause fragmentation of the molecular ions and thus allow for the determination of elemental compositions. In addition, LC-MS/MS can be used to identify metabolites [10, 13].

The third separation technique, CE, is suited for the separation of polar and charged compounds, as compounds are separated on the basis of their electrophoretic mobility. However, the repeatability of migration time in CE is very poor compared to that of the retention time in GC and LC. This factor is especially critical in metabolomics because a high accuracy of metabolite peak alignment must be achieved prior to downstream data analyses. Large variations in migration time make alignment very challenging [14].

MS-based metabolomics experiments using these separation techniques can be conducted using three approaches: 1) non-targeted metabolic profiling where the identity and relative quantity of as many metabolites as possible are obtained; 2) targeted profiling where the absolute quantity of a pre-selected smaller set of metabolites, typically related by chemical or biological similarity, are obtained using internal standards and reference compounds, and 3) metabolic fingerprinting where a global snapshot of the metabolism is acquired and compared without performing quantification and chemical identification. Biologically interesting components can then be subjected to targeted profiling for identification and quantification.

These aforementioned analytical platforms (LC-, GC, and CE-MS) and metabolomic approaches (profiling and fingerprinting) have been applied widely in metabolomics research. A number of excellent review articles have been published summarizing this work [3, 15–24]. Carrying out a metabolomics study generally involves a sequence of steps: experimental design, sample collection and preparation, analysis of samples on analytical platforms, and data handling. Among these steps, the last step relies heavily on bioinformatics. Because mass spectrometry-based metabolomics studies generally produce large and complex datasets, the bioinformatics involved is nontrivial and requires specialized computational algorithms and software tools.

For metabolic profiling where identification and quantitation (semi-quantitation for non-targeted and absolute quantitation for targeted profiling) are performed, bioinformatics includes three sequential steps: (1) data processing converts mass spectral data into tables of known or unknown metabolites with their identity and quantity, (2) data analysis identifies interesting metabolites or metabolic patterns through statistical analyses, clustering, and classification, and (3) data interpretation places the metabolomics data in the context of metabolic pathways and integrates metabolomics data with data from other omics platforms. Combination of the three steps is essential for knowledge discovery from the metabolomics experiments.

Among the three data handling steps, processing of raw mass spectral data is critical because any inaccuracy in this stage will propagate to the subsequent two steps. For a GC-MS based metabolomics study, data processing is even more critical due to co-elution of two or more compounds and the in-source fragmentation of molecular ions caused by the hard EI ionization. Co-elution and in-source fragmentation cause the resulting raw mass spectra to consist of mass peaks from all of the co-eluting metabolites. In order to identify and extract the quantitative information of the corresponding metabolites, the spectrum for each single metabolite has to be constructed based on the composite spectra. This spectrum construction step is called *deconvolution* in GC-MS data processing.

Considering the importance of GC-MS for analyzing compounds commonly observed in fruits, vegetables, nutritional and medicinal plants, and human biofluids after food digestion, we would focus on the deconvolution aspect of GC-MS data processing in this review. We will review the progress that has been made so far, and provide our thoughts on future developments that will enable further progress in metabolomics research. For the other aspects of data processing and the subsequent data analysis and interpretation, we refer readers to the research article by Stein el al. [25], review articles by Wishart [26, 27] and by Halket et. al. [10].

## Deconvolution

**Figure 1:**
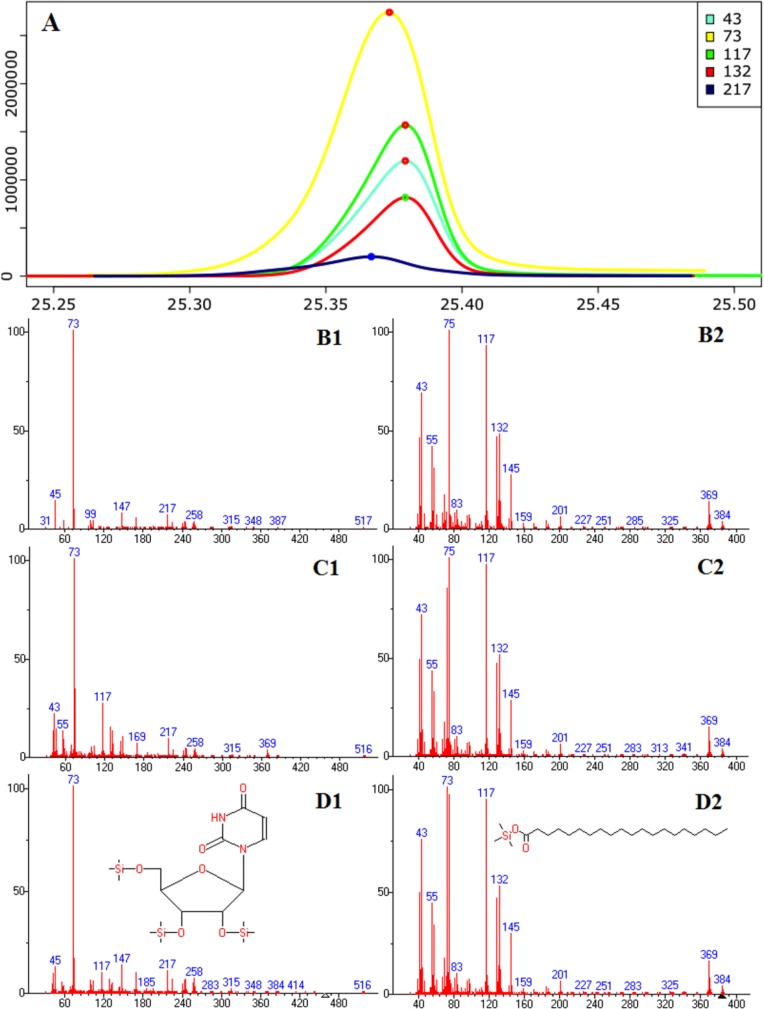
Comparison of constructed mass spectra and subsequent metabolite identification results with and without accurate deconvolution of shared peaks from two co-eluting compounds, uridine (Left) and n-Eicosanoic acid (Right). (**A**) Raw EICs of selected masses. Mass 43, 73, and 117 marked with red circles are shared by both compounds. Mass 217 is unique to uridine while mass 132 is unique to n-Eicosanoic acid. (**B1-2**) Constructed mass spectra of uridine (B1) and n-Eicosanoic acid (B2) after deconvolution using ADAP 1.0. The shared masses 43, 73, and 117 are only included either in the spectrum for n-Eicosanoic acid or in uridine. Their matching scores are 810 and 881, respectively. (**C1-2**) Constructed mass spectra after deconvolution that decomposes shared peaks. Each of the shared masses, 43, 73, and 117, is included in the spectra for both n-Eicosanoic acid and uridine. Their matching scores are 909 and 948, respectively. (**D1-2**) Reference spectra from an in-house library. This Figure is Figure 1 in the original article [33]. Reprinted with permission from the American Chemical Society.

For GC-MS data, deconvolution is the process of computationally separating co-eluting components and creating a pure spectrum for each component. Specifically, for each observed EIC that results from two or more components, deconvolution calculates the contribution of each component to the EIC. Figure 1 depicts the necessity of deconvolution.

Deconvolution had evolved based on the work of a number of researchers [28, 29] and was popularized with the publication of the AMDIS (Automated Mass Spectrometry Deconvolution and Identification System) algorithm [30] and subsequent development of the software tool [31]. The principle behind AMDIS also formed the basis for subsequent developments of other deconvolution algorithms including MetaboliteDetector [32] and ADAP-GC [33]. These three software tools are freely available. Commercial software tools have also been developed that include ChromaTOF [34] and AnalyerPro [35]. As far as we know, the technical details of the latter two software tools have not been released. Next we will examine the deconvolution algorithms implemented in AMDIS, MetaboliteDetector, and ADAP-GC.

### Spectrum deconvolution by AMDIS

The overall deconvolution process in AMDIS consists of four sequential steps: 1) noise analysis, 2) component perception, 3) model shape determination (The original paper included this step as part of step 2 [30]. For convenience of comparison with other algorithms in this review, we describe this step separately), and 4) spectrum deconvolution. The first step extracts the noise characteristics for a GC-MS data file by calculating the noise factor to be used for representing signal magnitude in noise units. Noise factor is conceptually defined as

1 $$N_{f}=\text{average random deviation}/\sqrt{\text{signal}}$$

**Figure 2:**
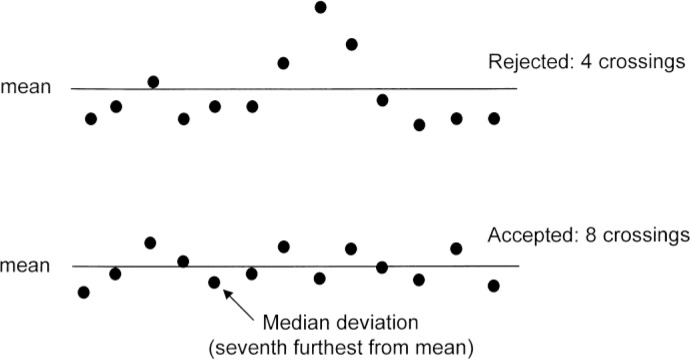
Illustration of the determination of the noise factor (N f) from 13- scan ion chromatogram segments. The upper chromatogram is rejected because it has fewer than seven “crossings” of the mean. The lower ion chromatogram crosses the mean eight times, so provides a sample noise factor. The *median* distance from the mean (seventh closest to the mean) is used to generate a sample noise factor N f. The final N f for the analysis is taken as the median of all sample values. This Figure is Figure 1 in the original article [30]. Reprinted with permission from Elsevier.

Briefly, each EIC and the total ion chromatogram (TIC) are divided into segments of a certain number of scans (13 scans were used in the original publication [30]). For each segment that has no zero abundance values, a *mean* abundance is computed and the number of times that this mean value is crossed is counted. If the number of crossings is greater than one-half of the number of scans (*i.e*., 7 scans when the segment is 13 scans wide), this segment is accepted and the *median* deviation from the mean abundance for that segment is found (Figure 2). This deviation is the average random deviation within this segment. It is then divided by the square root of the mean abundance for that segment to obtain a segment-specific *N*_*f*_ value as defined in Eq. 1. The *median* of all of the segment-specific *N*_*f*_ values is taken as the characteristic *N*_*f*_ value for the entire GC-MS data file. The square root of a signal multiplied by *N*_*f*_ is the magnitude of this signal in ‘noise units’.

**Figure 3:**
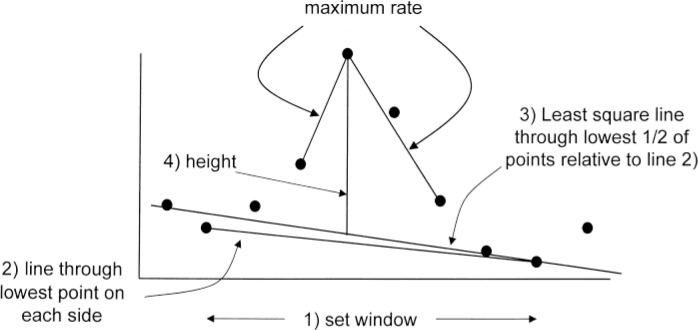
Four steps for determining whether an ion chromatogram peak is large enough to be used for peak perception. (1) A scan window is set using minima on each side of the peak; (2) a tentative baseline is drawn between the lowest points on each side (readjusted if a point between these end points falls below the line); (3) a least-squares line is drawn using the lowest one-half of points as measured from the baseline in step 2; (4) signal height between the maximum and least squares line is computed. Peaks must have heights larger than four noise units (N f v ) for use in peak perception (*A* is the absolute abundance at the peak maximum). This Figure is Figure 2 in the original article [30]. Reprinted with permission from Elsevier.

The second step, component perception, perceives individual chromatographic components. The rationale behind component perception is that a component exists when a sufficient magnitude of ions maximize together. It is achieved through a process as illustrated in Figure 3 where the deconvolution window is established first. When determining a deconvolution window, AMDIS “*sequentially examines scans starting at the scan of maximization and proceeds in the forward and reverse directions up to a pre-set maximum number of scans (12 is the default)*. *If a signal abundance is encountered that is more than five noise units greater than the smallest abundance between that scan and the starting scan (with noise units measured for the smallest abundance), then it is presumed that another component has been found and the window length is set to the preceding scan. Also, if the intensity falls below 5% of the maximum intensity, the window is fixed at that scan.”*

The third step determines the model peaks to be used in the next step for deconvolution. The model shape for each perceived component is taken as the sum of the individual ion chromatograms that maximize together and whose sharpness values are within 75% of the maximum value for this component. The sharpness value between the maximum abundance, *A*_*max*_, and an abundance value located *n* scans from the maximum, *A*_*n*_ is defined as:

2 $$\left(A_{\max}-A_{n}\right)/\left(n*N_{f}\sqrt{A_{\max}}\right)$$

The maximum sharpness values on each side of the maximum scan are averaged as the sharpness value of this ion chromatogram.

The last step, deconvolution, extracts ‘purified’ spectra from individual ion chromatograms for each component using the model shapes and the least-squares method. Briefly, each ion chromatogram is fit to the model profiles allowing a linear baseline:

3 A(n)=a+b*n+c*M(n)+d*Y(n)+e*Z(n)+K

This four-step process of deconvolution that AMDIS uses forms the basis of deconvolution in MetaboliteDetector [32] and ADAP-GC 2.0 [33]. Even though the deconvolution principle underlying these three algorithms are similar, the algorithms differ in details that we describe next.

### Spectrum deconvolution by MetaboliteDetector

**Figure 4:**
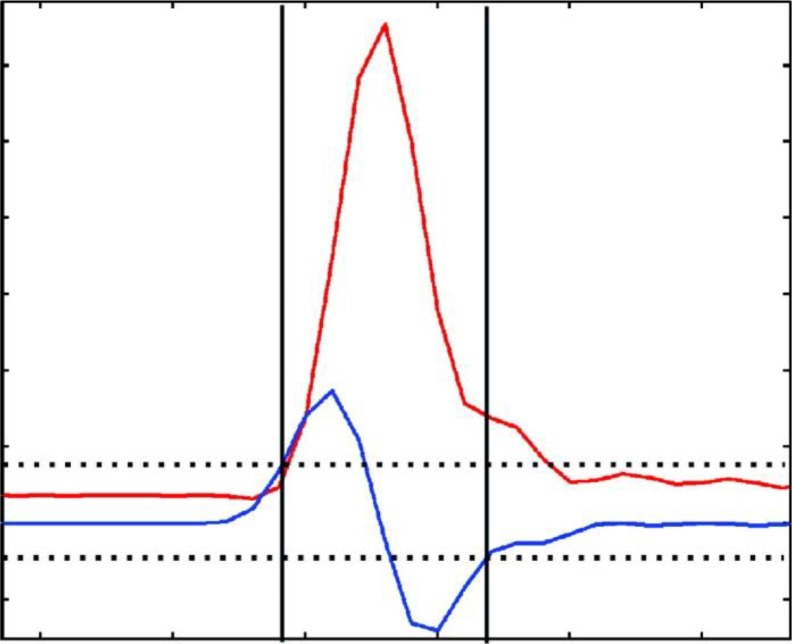
Single ion chromatographic peak detection. The peak borders are determined based on the first derivative of the intensity values. The red line represents the intensity values vs. retention time. The blue line depicts the first derivative of the intensity values. If the values of the first derivative cross the peak threshold, a peak begin or end is set (dotted lines). This Figure is Figure 2 in the original article [32]. Reprinted with permission from American Chemical Society.

Deconvolution in MetaboliteDetector differs from AMDIS in component perception and model peak determination. For component perception, it detects the beginning and ending of chromatographic peaks by calculating the first derivative of the smoothed intensity values (Figure 4):

4 $$f^{'}\left(x\right)=\frac{-2x_{-2}-x_{-1}+x_{+1}+2x_{+2}}{10}$$

A resulting peak is counted as valid if three criteria are met: the peak must consist of more than three values, the height above the baseline in signal-to-noise units of the maximum peak value must exceed a predefined threshold, and the quality of the peak shape must be in a certain range. The quality of a peak shape, named *discrepancy index*, is defined based on the assumption that all values of the first derivative of an *ideal* single peak must be positive from the peak beginning to the peak maximum and negative from the peak maximum to the peak ending. The absolute values of the first derivatives that agree with this assumption are summed as ideal slopes and the absolute values of the first derivatives that disagree with this assumption are summed as nonideal slopes. The discrepancy index *q*_*p*_ of a peak shape is formally defined as the ratio of the nonideal to ideal slopes:

5 $$q_{p}=\frac{\text{sum of nonideal slopes}}{\text{sum of ideal slopes}}\times 100\%$$

Reasonable values of *q*_*p*_ are in the range between 0% and 10%.

To determine the model peak shape for each perceived component, MetaboliteDetector sorts all single ion peaks of a compound having *q*_*p*_ values below 10% by their sharpness values. The top 25% of the peaks in terms of the sharpness value are summed to form the model peak for this compound.

### Spectrum deconvolution by ADAP-GC 2.0

**Figure 5:**
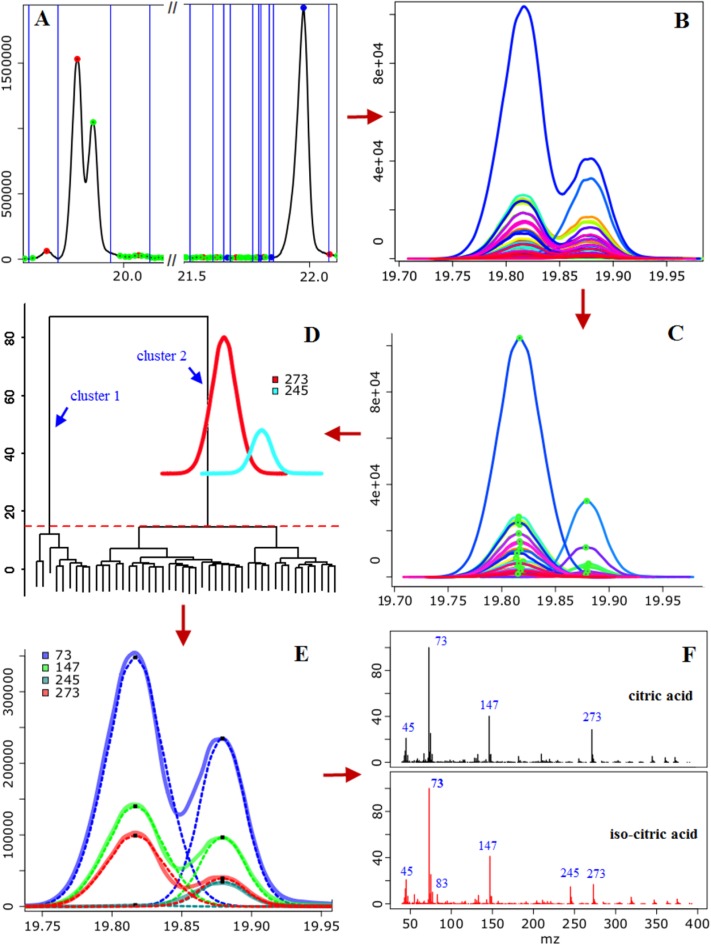
Illustration of the data analysis workflow of ADAP-GC 2.0 using two co-eluting compounds. (A) Detection of CPFs from TIC and determination of deconvolution windows (indicated by blue vertical lines). Two representative CPFs are displayed: one simple CPF marked by a blue solid circle at the apex and one composite CPF marked by red and green solid circles at the apexes. (B-F) Deconvolution of the EIC CPFs that have given rise to this composite TIC CPF. (B) Raw EICs of 46 good candidates. (C) The constructed mirror images of the 46 good candidates. (D) Determination of the number of components and corresponding model CPFs for each component using hierarchical clustering. The red dashed line indicates the empirical cutoff for determining the number of clusters. (E) The composite CPFs of masses 73, 147, 245, and 273 (solid line) were decomposed into simple CPFs (dashed line). (F) Two mass spectra were constructed and identified. This Figure is Figure 3 in the original article [33]. Reprinted with permission from the American Chemical Society.

Deconvolution in ADAP-GC 2.0 differs from AMDIS and MetaboliteDetector in terms of component perception, noise analysis, and model peak determination (Figure 5). Specifically, ADAP defined the concept of chromatographic peak features (CPFs).

A CPF is the elution profile of a minimum number of components that makes the elution profile complete, with ‘complete’ meaning that the elution profile lasts from the beginning to the end of the elution of the component(s). A CPF that results from a single component is defined as a *simple* CPF, and a CPF that results from two or more components is defined as a *composite* CPF. A simple CPF has only one local maximum, and a composite CPF could have one, two, or more local maxima. ADAP-GC 2.0 detects a CPF by determining the beginning, ending, and apex time of each local peak based on local maximum (for peak apex) and minimum (for beginning and ending). To determine if a peak is a simple CPF or part of a composite CPF, the algorithm calculates the ratio of intensity values at the boundaries to the intensity value at the peak apex. If one of the ratios is higher than a configurable threshold, this peak is considered part of a composite CPF. All of the neighboring incomplete peaks are then merged to form a composite CPF.

Subsequently, ADAP-GC 2.0 determines the deconvolution windows based on both the TIC and EIC. Basically, the beginning and ending of a TIC CPF delimit a window and any EIC CPF whose peak apex falls in the window will participate in the window-specific deconvolution. For each deconvolution window, ADAP determines the number of components and the model peak shape for each component. Unlike the model peak in AMDIS and MetaboliteDetector, ADAP-GC 2.0 defines a model CPF as the elution profile of a compound when it elutes from a chromatography system alone and its concentration is within the linear dynamic range of the mass spectrometer. As such, a model peak can result from the elution of a single component only. To determine the model CPF, ADAP first calculates five quality metrics of each CPF. These five metrics are sharpness, signal-to-noise ratio (SNR), peak intensity, Gaussian similarity, and the mass. The sharpness value of a CPF is calculated as

6 $$\text{sharpness}=\sum_{i=2}^{p}\frac{I_{i}-I_{i-1}}{I_{i-1}}+\sum_{i=p}^{N-1}\frac{I_{i}-I_{i+1}}{I_{i+1}}$$

Where *N* is the total number of time points for a CPF, *p* is the time index of the apex, and *I*_*i*_ is the abundance value at time index *i*. The SNR is estimated based on the high- and low-frequency signal components of the CPF, which is calculated using the continuous wavelet transform. For details about other metrics, please refer to the original article.

Following the calculation of the five metrics, ADAP selects those CPFs with high values of sharpness, SNR, and Gaussian similarity. Each of the filter-passing CPFs is assigned a total quality score calculated as

7 $$\text{total score}=\left({\text{c}}_{\text{1}}\right)\left(mass\right)+\left({\text{c}}_{\text{2}}\right)\left(\text{Gaussian similarity}\right)+\left({\text{c}}_{\text{3}}\right)\left(\text{apex intensity}\right)+\left(c_{4}\right)\left(\text{SNR}\right)$$

Those CPFs whose total score pass the following threshold are considered good candidates of model CPFs:

8 $$\text{total scor}{\text{e}}_{\text{threshold}}=\text{min}\left(\text{total scores}\right)+0.25\left[\text{range}\left(\text{total scores}\right)\right]$$

Finally, all of the good candidates for model CPFs participate in a hierarchical clustering process for ADAP to determine the most likely number of components in the current deconvolution window and the corresponding model peak for each component.

## Conceptual comparison of deconvolution algorithms in AMDIS, MetaboliteDetector, and ADAP-GC

Each of the aforementioned computational steps plays an important role in determining whether or not metabolic changes can be detected in metabolomics studies.

1. Noise analysis: Mass spectra and chromatograms that are obtained from the spectra are inherently noisy. All of the three algorithms we described above incorporated noise analysis. AMDIS and MetaboliteDetector calculate the noise factor of a GC-MS data file and then convert the signal magnitude in noise units in some of the subsequent calculations. ADAP-GC 2.0 uses a completely different approach by directly computing the signal-to-noise ratio of each CPF and uses it as a filter to prevent noisy CPFs from being selected as model CPFs. Without direct comparison between these two approaches, it is challenging to state which one leads to better performance.
2. Determination of deconvolution windows: AMDIS's approach often causes deconvolution windows to be narrower than optimal for quantitation, as pointed out in the original article [30]. This happens in two scenarios where a deconvolution window contains only part of the entire chromatographic peaks. One is when a chromatographic peak is very intense, the deconvolution window is fixed at 5% of the maximum intensity, and the remaining part of the peaks that is below 5% of the maximum intensity is outside of the deconvolution window. If neighboring co-eluting components are in very low concentrations, their abundance will be much lower than that of the dominating peak and most of their chromatograms will be outside of the deconvolution window. We have observed many examples of this case in our own work. The other scenario is when the algorithm determines that a co-eluting component is encountered because the abundance value is more than five noise units greater than the smallest abundance between that scan and the maximization scan. Since the window is set to the preceding scan, part of the peak will be left out of the deconvolution window as well.
  In order to resolve this issue, ADAP-GC 2.0 tries to detect composite CPFs and ensure that their deconvolution windows contain the entire CPF. Conceptually, this should increase the accuracy of the quantitative information that ADAP extracts about metabolites from the data.
  In MeteboliteDetector, how deconvolution windows are determined was not explicitly described. Regardless of the specific approach, it must utilize the information about the beginning and ending of chromatographic peaks. MetaboliteDetector determines the beginnings and endings using first derivatives as in Eq. 4. For details, please refer to the original article. This approach tends to be very sensitive to noise because the derivative operation amplifies noise [36]. Even though chromatograms are smoothed prior to this step, fluctuations can still exist along a chromatographic peak and cause the algorithm to decide that a beginning or ending of the chromatographic peak has been encountered. Consequently, part of the peak will be left out of the deconvolution window and the quantitative information extracted from the data about the corresponding component will be reduced.
3. Determination of model peaks: The central goal of deconvolution is to decompose a composite CPF into the weighted summation of the model peaks and then form the spectrum of a single component based on the weights in Eqn. 3. Specifically, the resulting c * M (*n*max) in Eqn. 3, where *n*max denotes the scan with the maximum model peak abundance, will be used as the abundance for the corresponding *m/z* in the extracted spectrum. Conceptually, it should be preferred that each model peak corresponds to one single component only. However, since both AMDIS and MetaboliteDetector use a summation of peaks as the model peak and one or more of the constituent peaks could consist of signals from multiple co-eluting components, the likelihood that the final model peak also consists of signals from multiple co-eluting components is very high. Consequently, the magnitude of the mass peaks in the extracted spectrum will be inaccurate, which could ultimately cause both false positive and false negative metabolite identifications. Moreover, the quantitative information for corresponding components will be inaccurate as well since it is calculated based on the extracted spectrum.
  ADAP, however, selects only the purest peak for model peaks by using five peak quality metrics. On the other hand, ADAP has a weakness too in determining model peaks. It favors model peaks that are symmetric and resemble a Gaussian curve. In reality, fronting and tailing do occur and cause asymmetric peak shapes even when a compound elutes alone from the chromatography system. This issue can conceptually be alleviated or eliminated by decreasing the weighting factor that is assigned to the Gaussian similarity in Eq. 7. Of course, testing is needed to check if the overall performance of the algorithm is affected as a result of this change.

Based on the above description, we can see that it will be worthwhile to carry out a detailed comparison about the performance of the critical computational steps and come up with the best overall deconvolution strategy. Even though the aforementioned three algorithms have not been directly compared, a comparison between AMDIS and two commercial software packages, ChromaTOF [37] and AnalyzerPro [35], has been performed by Lu et. al [12]. The article concluded that none of these approaches provided a comprehensive solution meeting the specific needs of metabolomics [12]. Specifically, 1) AnalyzerPro tends to produce a great number of false negatives, 2) ChromaTOF and AMDIS tend to produce multiple peak assignments that clearly correspond to a single chromatographic peak and chemical entity, and 3) all of them are still fairly slow for the flood of data from high-throughput metabolomics studies. Since details about the deconvolution algorithms in ChromaTOF and AnalyzerPro are unknown, we are unaware of the possible factors that could have caused their respective issues.

For AMDIS, it was originally developed for automated identification of chemical weapons and related compounds [30]. Therefore, there is a strong emphasis in AMDIS on low false negative rates for metabolite identification. Since identification is achieved by matching the ‘purified’ spectra for single components that are obtained from deconvolution against libraries of reference mass spectra, each step of the deconvolution process needs to ensure that as few components as possible are missed. As a result, AMDIS is best suited for analyzing simple mixtures consisting of a small number of compounds. When analyzing complex mixtures, time-consuming manual checking of the results is necessary in order to reject false positive identifications [32].

Appropriate configuration of processing parameters can help alleviate the challenge to find an acceptable balance between false positive and false negative identifications. However, without prior knowledge about the sample composition in un-targeted metabolic profiling experiments, researchers usually do not know what parameter setting is appropriate for their datasets. This issue exists in other algorithms as well.

## Summary and Outlook

The past decade has witnessed tremendous progress on metabolomics bioinformatics research. However, the progress has not been as fast as that on the instrumentation side as mass spectrometry coupled to chromatography is becoming increasingly sensitive and their operations are becoming increasingly high-throughput. Next, we provide our thoughts on new bioinformatics capabilities for GC-MS data processing that are needed for metabolomics to progress further.

1. More efficient and reliable deconvolution algorithms need to be developed for identification and quantification of metabolites due to the aforementioned limitations of existing deconvolution algorithms. Newly developed algorithms need to be implemented into user-friendly and high-throughput software tools. These software tools should be equipped with visualization capabilities that will allow metabolomics researchers to visually examine intermediate and final results for verifying the correctness of significant metabolites detected in the data analysis and data interpretation stages. Development of these algorithms and software tools will benefit greatly from an interdisciplinary team of researchers and software engineers with engineering, chemistry, math, and computer science background.
2. Computational algorithms and software tools are also needed for identifying unknown metabolites. This is because, with unprecedented sensitivity, dynamic range, and throughput, both GC-MS and LC-MS analytical platforms can now detect many metabolites in a short time frame that could not be observed before. However, the power of the detection methods has outstripped our ability to process the data. Often, the number of unidentified metabolites in a sample is 2-3-fold more than the number of identified metabolites even after extensive data processing. This is because even the largest and most comprehensive of currently available libraries contain only a small portion of the endogenous metabolites found in biological samples [38–41].

Identifying these unknown metabolites is currently a major bottleneck in metabolomics [42, 43]. This issue is even more acute in studies investigating plant products because diverse plant species are estimated to produce more than 200,000 metabolites of enormous biochemical diversity of which only 10,000 chemical structures are known [4, 11]. Even the extensively studied model plant *Arabidopsis thaliana* has a large number of enzymes whose substrates and products remain unknown [44]. Meanwhile, commercially available standard reagents, especially those of secondary metabolites produced by plants, are very limited in number. Given the fundamental importance of biochemicals to agriculture, nutrition, and health, the potential benefit of identifying even half of these unknown metabolites is astronomical.

Therefore, a pressing need in the metabolomics community is the development of effective methods for prioritizing, studying, and ultimately identifying uncharacterized metabolites [40]. To address this need, novel bioinformatics capabilities to process raw mass spectral data and to create libraries of unidentified spectra must be developed [10, 11, 26, 38, 45, 46].

For GC-MS-based metabolomics, some progress has been made in the effort to use and catalogue unidentified GC-MS spectra in metabolomics studies [4, 37, 38, 46–64]. Among these efforts, most rely on nonsystematic, low-throughput, manually assisted curation of unidentified peaks [38], and only a few use more advanced approaches with minimal manual annotation. These latter efforts include the work on developing the volatile compound mass spectral database named vocBinBase [63], developing a spectral library of unknown compounds for urine [46], and identifying conserved metabolites [38]. These three projects used spectra extracted from the raw mass spectral data by AMDIS or ChromaTOF (LECO Corp., Michigan, USA). In constructing the vocBinBase database, unknown spectra are filtered and only the filter-passing spectra are imported into the database. Unfortunately, since each spectrum in the database is obtained from only a single sample, it is very likely that it is not the best representation of the corresponding compound. Missing, inaccurate, or false peaks in these spectra will cause future false positive and false negative identifications, especially as the database grows and different compounds with similar spectra are incorporated into the library. A better approach is to construct a consensus spectrum from many measurements of the same compound. Consensus spectra are indeed used in some of these developments [38]. However, inaccuracies in these spectra still need to be corrected based on analyses of extracted spectra from multiple samples. This step is very important because inaccuracies are very likely to occur due to factors such as co-elution, low signal-to-noise ratios in some regions of the chromatograms, and inappropriate parameter settings in the process of chromatographic peak picking and deconvolution [12]. This spectra correction step will significantly increase our confidence in the consensus spectra. In addition, it will improve the accuracy of the quantitative information about the corresponding unknown metabolites, which is critical to comparative metabolomics

In addition to the above approaches, the widely used GOLM GC-MS mass spectral database incorporated mass spectra of unidentified metabolites. However, similar to the vocBinBase database, no derivation of consensus spectra or determination of the best representative spectra was implemented [65]. For LC-MS-based metabolomics, the creation of multiple databases of LC-MS/MS mass spectra has made significant contributions to the field, including METLIN [13] and MassBank [66]. However, there has been no report of systematically creating spectral libraries of unknown compounds.

This lack of capabilities to handle unidentified GC-MS and LC-MS spectra reveals that metabolomics bioinformatics is lagging behind technological advances in analytical instrumentation, and this is hindering investigators from taking advantage of data that the instruments already capture.
